# Gene Expression Factors Associated with Rubella-Specific Humoral Immunity After a Third MMR Vaccine Dose

**DOI:** 10.3390/v17091154

**Published:** 2025-08-23

**Authors:** Lara I. Teodoro, Iana H. Haralambieva, Inna G. Ovsyannikova, Krista M. Goergen, Diane E. Grill, Gregory A. Poland, Richard B. Kennedy

**Affiliations:** 1 Mayo Clinic Vaccine Research Group, Mayo Clinic, Rochester, MN 55905, USA; teodoro.lara@mayo.edu (L.I.T.); haralambieva.iana@mayo.edu (I.H.H.); ovsyannikova.inna@mayo.edu (I.G.O.); poland.gregory@mayo.edu (G.A.P.); 2 Department of Quantitative Health Sciences, Mayo Clinic, Rochester, MN 55905, USA; goergen.krista@mayo.edu (K.M.G.); grill@mayo.edu (D.E.G.)

**Keywords:** rubella, rubella vaccine, MMR, immunity, humoral immunity, gene expression, NGS, RNA, transcriptome, genetic markers

## Abstract

Rubella is typically a mild viral illness, but it can lead to severe complications when contracted during pregnancy, such as pregnancy loss or developmental defects in the fetus (congenital rubella syndrome). Therefore, it is crucial to develop and maintain protective immunity in women of childbearing age. In this study, we assessed the transcriptional factors associated with rubella-specific immune outcomes (IgG binding antibody and avidity, neutralizing antibody, and memory B cell ELISpot response) following a third MMR vaccine dose in women of reproductive age to identify key factors/signatures impacting the immune response. We identified baseline (Day 0) and differentially expressed (Day 28–Day 0) genes associated with several RV-specific immune outcomes, including the transferrin receptor 2 (*TFR2*), which is an important factor regulating iron homeostasis and macrophage functional activity, and a close functional homolog of TFR1, the cellular receptor of the New World hemorrhagic fever arenaviruses. We also identified enriched KEGG pathways, “cell adhesion molecules”, “antigen processing and presentation”, “natural killer cell-mediated cytotoxicity”, and “immune network for IgA production”, relevant to immune response priming and immune activation to be associated with RV-specific immune outcomes. This study provides novel insights into potential biomarkers of rubella-specific immunity in women of childbearing age.

## 1. Introduction

Rubella is considered a mild viral illness characterized by fever, maculopapular rash, and lymphadenopathy, with or without joint involvement (arthritis and arthralgia are noted mostly in adult women) [1]. However, the disease in pregnant women frequently results in pregnancy loss or has detrimental consequences for fetal development, resulting in deafness and ocular, cardiac, and other congenital abnormalities, known as congenital rubella syndrome (CRS) [1]. While the global burden of CRS decreased 66% (from 105,000 cases in 2010 to 32,000 cases in 2019) [2,3], if MMR vaccination rates drop below the herd immunity threshold (83–85%) [4], it will raise concerns about the re-emergence of the disease [5]. The tropism of rubella virus is not fully elucidated, but the virus infects and replicates in the nasopharynx/upper respiratory tract, spreads to the regional lymph nodes, and during systemic viremia in pregnant women crosses/infects the placenta and fetal tissues, including brain tissue (CRS) [6]. Of the immune cells, it is established that rubella virus preferentially infects macrophages, neutrophils, the microglia (the brain resident macrophage cells), and possibly monocytes and dendritic cells [6,7], which may contribute to virus dissemination and host innate/inflammatory immune response and influence rubella virus-specific adaptive immunity.

Rubella vaccination is considered effective and confers protection in 95% of vaccinated individuals after a single dose [1]. There is substantial inter-individual variability in immune responses to rubella, even among immunocompetent individuals with one or two documented doses of a rubella-containing vaccine. Those with suboptimal responses are more likely, over time, to experience waning immunity and fall below the threshold of 10 IU/mL, the established cutoff for defining rubella immunity [8,9,10,11]. In a large study comprising a highly immunized population from Olmsted County, MN, and surrounding areas (N = 1393 subjects, 20 to 44 years of age, 80.2% females), we have reported 2.2% seronegative individuals, a relatively small but sizable percentage [8]. Such seroepidemiological data suggest a small but potential risk for infection and pregnancy complications for women of reproductive age upon rubella wild-type virus exposure. Evaluation of the relevant genetic and transcriptional factors and immunological characteristics of rubella vaccine hyporesponsiveness/waning immunity in vulnerable populations (and other populations) is warranted to identify individuals at higher risk of acquiring the disease and unveil the mechanisms, which might result in long-lasting and robust vaccine response [12,13,14,15,16].

Here, we report the results of a longitudinal vaccine study in 98 women of reproductive age, considering their rubella-specific baseline immunity and the change in immune response after a third dose of MMR vaccine. The study subjects were chosen from those with residual rubella-specific antibody titers after prior MMR doses in the top and bottom 30th percentile of the baseline antibody response measured in a larger cohort (n = 1117) from the local community [8]. We conducted longitudinal gene expression profiling before and after administration of a third MMR vaccine dose to elucidate the potential factors and mechanisms that may be associated with and/or mediate changes in rubella immune response following a third vaccination.

## 2. Materials and Methods

The study cohort, rubella IgG and neutralizing antibody assays, memory B cell ELISpot assay, and RNA extraction methods have been thoroughly described in our previous publications [8,10].

### 2.1. Study Cohort

The study cohort has been described in detail by Haralambieva et al. (2020) [10]. Our analysis included 98 healthy female participants aged 30.7–40.4 years from Olmsted County, MN, USA and nearby areas, who were enrolled at the Mayo Clinic in Rochester, MN, USA and had documented receipt of two previous MMR vaccine doses. Individuals were selected based on rubella virus (RV)-specific antibody IgG titers measured by enzyme-linked immunosorbent assay (ELISA) from a pool of 1117 serum samples collected through the Mayo Clinic Biobank. The cohort represented those in the top and bottom 30% of the baseline IgG titer distribution. Blood samples were obtained at baseline (prior to vaccination) and on Day 8 and Day 28 following administration of a third MMR dose. Written informed consent was obtained, and all study procedures, including those related to the Mayo Clinic Biobank, were approved by the Mayo Clinic Institutional Review Board (IRB #15-007916).

### 2.2. Rubella Neutralizing Antibody Assay

Neutralizing antibody titers against RV were measured as previously described [10]. Neutralizing titers (NT_50_) were reported using the Karber method, representing the highest serum dilution at which the viral signal was reduced by at least 50% across the dilution series [10]. The assay demonstrated an intra-class correlation coefficient (ICC) of 0.89 based on log-transformed NT_50_ values from repeated measurements [10].

### 2.3. Memory B Cell ELISpot Assay

The frequency of RV-specific memory-like IgG B cells was measured in peripheral blood mononuclear cells (PBMCs) at baseline and Day 28 after administration of a third MMR vaccine dose. Quantification was performed using the Mabtech ELISpot^PLUS^ kit for human IgG (Mabtech Inc.; Cincinnati, OH, USA), following the manufacturer’s instructions. Prior to analysis, PBMCs/B cells underwent non-specific in vitro pre-stimulation for three days with human recombinant IL-2 and Toll-like receptor (TLR) agonist R848. ELISpot plates were coated with RV antigen (HPV77 RV strain) obtained from Meridian Life Science Inc. (Memphis, TN, USA). The frequency of antigen-specific memory B cells was expressed as spot-forming units (SFUs) per 2 × 10^5^ cells, calculated as the median of quadruplicate RV-specific responses after subtracting subject-specific background values (no-antigen control). The assay showed strong reproducibility, with an average intra-class correlation coefficient (ICC) of 0.88 across replicate measurements.

### 2.4. Gene Expression

Gene expression in response to a third MMR vaccination dose was assessed in PBMCs collected at baseline and Day 28 post-vaccination. Briefly, mRNA was extracted using the Qiagen RNeasy Plus Mini Kit (Qiagen, Valencia, CA, USA) following cell preservation in RNAProtect (Qiagen, Valencia, CA, USA). All RNA and cDNA samples passed a quality control on an Agilent 2100 Bioanalyzer (Agilent; Palo Alto, CA, USA). Libraries were prepared and sequenced on the Illumina HiSeq 4000 platform (101 bp paired-end reads, 9 samples/lane) at Mayo Clinic’s Advanced Genomics Technology Center. Raw paired-end RNA sequencing reads were processed using the MAP-RSEQ version 3.0 pipeline, aligned to the hg38 reference genome using STAR, and quantified with featureCounts [17,18], using Ensembl v78 annotations.

Gene expression data were processed and normalized as previously described [19]. Briefly, conditional quantile normalization was applied, and only genes classified as protein-coding or annotated as processed pseudogenes were retained for analysis. To filter out low-abundance transcripts, genes were excluded if their coefficient of variation fell below the 25th percentile or if the median difference in expression between RV-stimulated and unstimulated cells was under 16. For the remaining 12,925 transcripts, expression values were normalized by centering on the mean and scaling according to standard deviation.

### 2.5. Statistical Analysis

Demographic and immune response variables were summarized using medians and the first (Q1) and third (Q3) quartiles.

For analytical purposes, we defined the response to a third dose of MMR as the change in antibody measurement from Day 0 to Day 28 (i.e., the Day 28 value minus the Day 0 value). To explore this relationship between humoral immunity and gene expression, we modeled three response variables: (1) the change in RV-specific IgG titer, (2) the change in neutralizing antibody titer, and (3) the Day 28 memory B cell ELISpot response (estimating RV-specific memory B cell frequencies) at Day 28. Since the ELISpot response is returned as counts, we fit a Poisson regression model, adjusting for batch effects. The residuals from this model were used as the response variable.

Elastic-net linear regression (α = 0.9 with 10-fold cross validation) was used to identify genes associated with each endpoint. Response variables and gene expression values were standardized to allow for within-model comparisons of the regression coefficients. Analysis was conducted using the glmnet package in R version 4.2.2 [20].

Over-representation analysis using KEGG pathways was run using the enrichKEGG function in the R cluster Profiler package [21]. The *p*-value cutoff was 0.05, and adjusted *p*-values were calculated using the Benjamini–Hochberg method.

## 3. Results and Discussion

### 3.1. Demographic and Immune Response Characterization of Study Subjects

This analysis included 98 females with a median age at enrollment (third MMR dose) of 35.2 years. The median time from the second rubella vaccination to study enrollment was 23.2 years (IQR: 18.7–25.5), as summarized in Appendix A, Table A1. Of the 98 study subjects, 53 were recruited as a low-antibody group and 45 as a high-antibody group according to prior screening of RV-specific IgG antibody titers [8]. Most study participants identified as White/Non-Hispanic or Latino. Demographic and clinical characteristics were balanced between the groups. Immunological outcomes (IgG titer, neutralizing antibody titer, avidity index, and memory B cell frequencies) at baseline and Day 28 post-vaccination have been previously described [10] and are summarized in Appendix A, Table A2. Consistent with other MMR vaccine studies [22,23,24], the subjects with a lower baseline antibody titer responded better to the third MMR vaccine dose, compared to those with higher antibody titer. In our study, the change in RV-specific IgG titers and avidity indexes from baseline to Day 28 was significantly greater in the group with low screening antibody titers (median change in IgG: 95.1 in the low vs. 49.3 in high, *p* < 0.001; median change in avidity index: 11.2 in the low vs. 5.6 in the high, *p* < 0.001), whereas in our study the change in neutralizing antibodies and RV-specific memory B cell frequencies did not differ significantly between groups [10].

### 3.2. The Impact of Baseline Gene Expression on Rubella-Specific Immune Outcomes

We used elastic-net linear regression models to identify baseline and differentially expressed (Day 28–Day 0) genes associated with rubella-specific immune outcomes following a third dose of MMR vaccination. The immune outcome of interest in these analyses was the change (Day 28–Day 0) in the immune response (IgG titer, neutralizing antibody titer, avidity index) after vaccination to account for pre-existing immunity (baseline immunity). The only exception was the RV-specific memory B cell response measured by ELISpot, where the Day 28 measure was included in the model due to the minimal detectable baseline counts.

**Figure 1:**
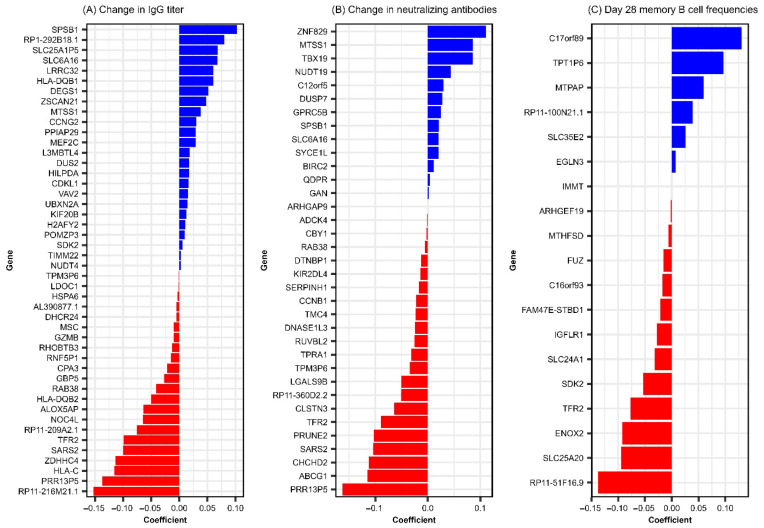
Elastic net linear model coefficients for baseline genes predictive of rubella-specific humoral response following MMR vaccination. This figure illustrates linear regression coefficients results from GLMNET modeling and summarizes the relative contribution of genes to RV-specific immune outcomes. (**A**) baseline genes predictive of change (Day 28–Day 0) in rubella IgG titer. (**B**) baseline genes predictive of change (Day 28–Day 0) in neutralizing antibodies. (**C**) Baseline genes predictive of Day 28 rubella-specific memory B cell frequencies.

Interestingly, we observed significant association between many baseline genes (predictors) on the antibody outcomes and/or the Day 28 memory B cell frequencies after vaccination, while the models assessing differentially expressed (Day 28–Day 0) genes produced limited results. We identified 46 baseline genes predictive of IgG response, 35 predictive of neutralizing antibody response, and 19 predictive of Day 28 memory B cell frequency. Baseline predictor genes associated with RV-specific immune outcomes are presented in Table 1, and model coefficients (mc) are illustrated in Figure 1. Notably, nine genes overlapped across at least two of these outcomes, as described in Table 2. No baseline genes were identified as being associated with changes in avidity index.

Of the identified predictors, we consider *TFR2* (transferrin receptor 2) to be the most consistent finding, since it emerged as a baseline predictor of rubella-specific immune response outcomes across both antibody immune response (RV-specific IgG titers, mc = −0.099 and neutralizing antibody titers, mc = −0.089, Figure 1 and Table 1) and rubella virus-specific memory B cell ELISPOT response (mc = −0.077, Figure 1 and Table 1). Interestingly, *TFR2* was also identified in the model of (Day 28–Day 0) gene expression, exhibiting a negative association with Day 28 memory B cell frequencies, albeit with a smaller effect (mc = 0.0031) on the immune outcome. *TFR2* encodes a protein involved in iron homeostasis, erythrocyte differentiation, and inflammatory response. It is preferentially expressed in the liver, muscle, lung, spleen, bone marrow, erythroid progenitors, neurons, microglia, and macrophages, regulating their polarization and functional activity (including cytokine and chemotactic factor production, reactive oxygen species/ROS production, antigen presentation, Toll-like receptor, and interferon signaling) [25,26]. Importantly, *TFR2* has close structural similarity and is functionally related to *TFR1. TFR1* has been identified as a bona fide cellular receptor, mediating the binding and entry of New World arenaviruses linked to hemorrhagic fever [27]. It is also suggested to facilitate the entry of other viruses, including influenza A, hepatitis C, and rabies [28,29,30]. Given this background, it is tempting to speculate that TFR2 may play a role in rubella virus binding and entry, potentially influencing viral cell entry, replication, antigen abundance, and immune response priming. We recognize that this is a working hypothesis that will require experimental validation. Our results point to a negative association between baseline gene expression and vaccine-induced rubella immune outcomes. This is counterintuitive but can be explained by the viral direct targeting of immune cells (e.g., macrophages, neutrophils, DCs) [6,7], which may directly impact innate/inflammatory response and skew/suppress cell immune function (e.g., antigen presentation, cytokine production, adaptive immune response priming), leading to blunting of adaptive immune response [26,31]. It has been demonstrated that lower CCR5 expression on CD4+ T cells is associated with improved immune responses to HIV [32]. This hypothesis is also supported by evidence that conserved linear structural motifs in viral proteins of different viruses can enable receptor binding and exploitation of host cellular machinery. For example, rubella virus E1 has been reported to contain epitope regions that align with those in measles virus [33,34,35], and its main antigen/target of neutralizing antibody response has structural similarity with the class II fusion proteins of alphaviruses, flaviviruses, and paleoviruses, despite minimal sequence homology [36,37]. These structural parallels suggest that shared binding motifs across unrelated viruses may enable attachment to similar cellular receptors, such as those in the transferrin receptor family, and promote viral entry. Further investigation and in-depth functional studies are necessary to reveal the role of *TFR2* in regulating rubella virus-specific humoral outcomes.

Among the 46 baseline genes predictive of change (Day 28–Day 0) in rubella IgG titer following a third MMR dose, 8 (*MTSS1*, *RAB38*, *PRR13P5*, *SARS2*, *SPSB1*, *TPM3P6*, *SLC6A16*, and *TFR2*) were also predictive of change in neutralizing antibody titer, manifesting positive (*MTSS1*, *SPSB1*, *SLC6A16*) or negative (*RAB38, PRR13P5, SARS2, TMP3P6, TFR2*) associations with immune outcome (Table 2) Moreover, these genes exhibited consistent coefficient directions across both models, as demonstrated in Table 2 and Figure 1A,B, suggesting shared transcriptional determinants of binding and functional/neutralizing antibody responses and highlighting potential baseline biomarkers of rubella antibody responsiveness following a third dose of MMR. Interestingly, a recent study analyzing RNA sequencing data from identical twins discordant for autism spectrum disorder identified *PRR13P5* as one of the differentially expressed genes and found enrichment in pathways related to immune cell signaling and immune response, suggesting a potential link between this gene’s expression and humoral immunity function [38]. Due to the limited understanding of *PRR13P5* (a gene negatively associated with rubella virus-specific humoral immunity in our study) function, further research is necessary to elucidate its role in immune regulation and potential impact on humoral response.

**Figure 2:**
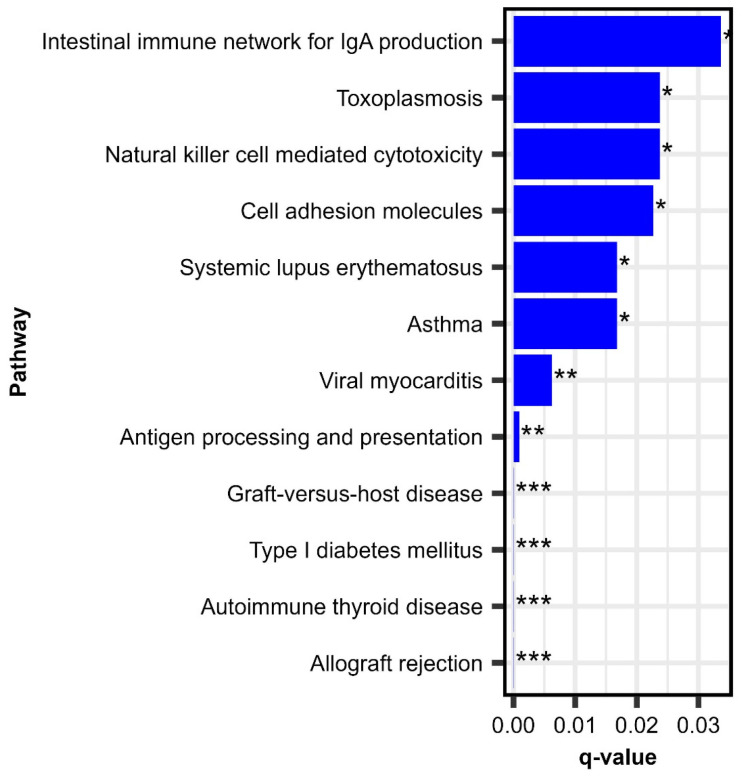
Enriched pathways of baseline gene expression associated with change (Day 28–Day 0) in rubella IgG antibody titer. This figure depicts *q*-values from KEGG pathway enrichment analysis on baseline genes associated with change (Day 28–Day 0) in RV-specific IgG antibody titer following a third dose MMR. * Adjusted *p*-value < 0.05; ** adjusted *p*-value < 0.01; *** adjusted *p*-value < 0.001.

We performed KEGG pathway over-enrichment analysis to identify relevant pathways and immune function-related cellular activities impacting the RV-specific antibody response following MMR vaccination. This analysis revealed 12 pathways that were significantly enriched and associated with antibody titer, illustrated in Figure 2. Four enriched pathways, “antigen processing and presentation,” “natural killer cell-mediated cytotoxicity,” “intestinal immune network for IgA production,” and, importantly, “cell adhesion molecules,” reflected pertinent biological processes/immune activity related to the viral attachment/cell entry and initial priming of immune response upon vaccination.

Of these, “antigen processing and presentation” is fundamental to the activation of CD4+ and CD8+ T cells, key factors in adaptive immunity and B cell help [39]. Simultaneously, natural killer (NK) cell-mediated cytotoxicity represents a crucial early defense, targeting infected cells and producing cytokines such as IFN-γ, which augment T cell activation/function [40]. Cell adhesion molecules can mediate attachment of viruses and facilitate the migration and interaction of immune cells, ensuring efficient trafficking to lymphoid tissues and stable contact between T cells and antigen-presenting cells [41]. Finally, the intestinal immune network for IgA production reflects mucosal immune activation and/or immunoglobulin production in general, potentially contributing to viral neutralization and the prevention of viral dissemination [42]. Collectively, these pathways highlight the coordinated activation of innate and adaptive immunity in response to MMR vaccination, underlining the biological mechanisms that drive effective priming and long-term protection against rubella.

It is interesting to compare our rubella findings with the findings from other vaccine and/or transcriptional studies, in particular with measles and mumps. Comparative gene expression studies of dendritic cells infected with MV vs. other pathogens (but not rubella virus) have demonstrated an MV-specific pronounced effect on the regulation of antigen presentation and innate antiviral immunity [43], consistent with our rubella study findings. Transcriptomic studies in measles vaccine recipients have emphasized the importance of plasma cell survival factors (e.g., *CD93* expression), chemokine and cytokine activity, cell adhesion, and cell migration for neutralizing antibody response to vaccination [44] and have identified early B cell transcriptomic signatures (*IL20RB*, *PMAIP1, BEX2*, *FAIM*, and *IL16* contributing to the selection of high-affinity B cells and the control of apoptotic pathways) that impact MV-specific antibody response after MMR vaccination [45]. Interestingly, studies exploring the influence of host genetic factors on immune response to measles and mumps vaccine have highlighted the critical roles of polymorphisms/genes linked to viral entry and innate/inflammatory pathways in governing antiviral immunity, as in our rubella vaccine study. A genome-wide association study demonstrated that polymorphisms in the measles virus receptor-encoding *CD46* gene and in *IFI44L* contribute to inter-individual differences in neutralizing antibody response following live measles vaccination [46]. Several studies have established that variance in the 19q13 genomic region (including the glycosyltransferase *FUT2* gene involved in host glycosylation and the sialic acid-recognizing receptors *SIGLEC5/SIGLEC14*) may influence mumps virus susceptibility/entry and contribute to cellular and inflammatory responses following mumps vaccination [47,48,49]. Thus, research on measles and mumps (both members of the Paramyxoviridae family) has identified both overlapping and distinct factors, genes, biological processes, and pathways regulating antiviral immunity in comparison to our findings with rubella.

### 3.3. The Impact of (Day 28–Day 0) Gene Expression Change on Rubella-Specific Immune Outcomes

**Figure 3:**
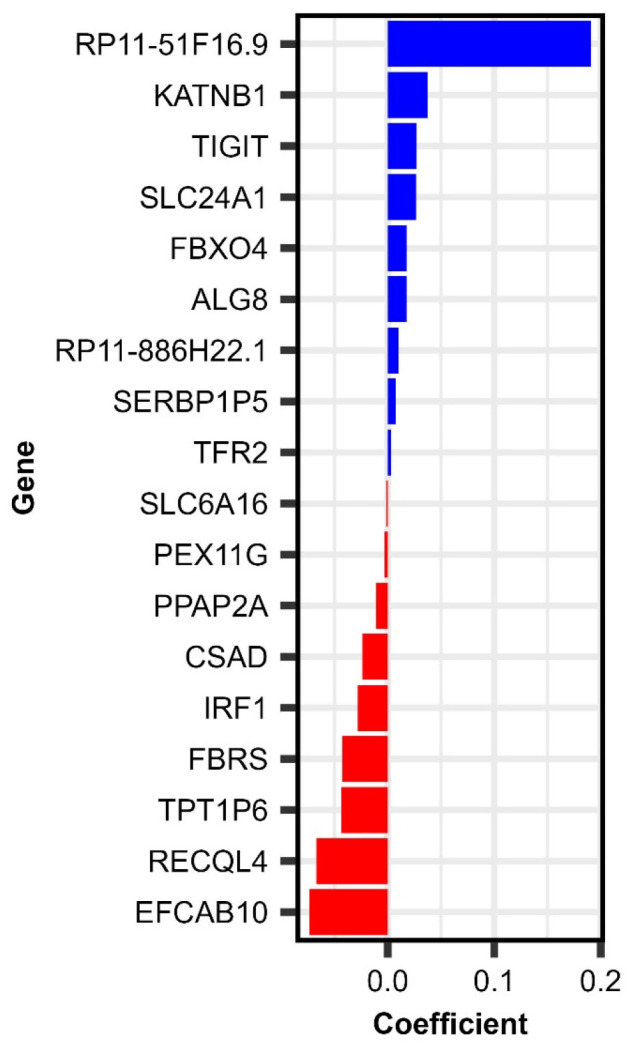
Elastic net linear model coefficients for (Day 28–Day 0) genes associated with rubella-specific Day 28 memory B cell frequencies following MMR vaccination.

These modeling efforts identified 18 differentially expressed (Day 28–Day 0) genes associated with Day 28 memory B cell ELISpot frequencies (RV-specific), depicted in Table 3 and Figure 3.

Summarizing our results from the (Day 28–Day 0) gene expression and baseline gene expression modeling by relevance, we identified four genes (*TFR2*, *SLC24A1*, *TPT1P6*, and *RP11-51F16.9*) consistently impacting several rubella-specific immune outcomes following vaccination, including the memory B cell frequencies, which are crucial for recall immune response upon subsequent viral exposure [50]. These genes, including *TFR2*, may serve as transcriptional markers of recall immune response, with potential implications for long-term humoral immunity and RV-specific memory B cell frequencies following MMR vaccination.

*SLC24A1* expression (Day 28–Day 0) change was positively associated with the immune outcome (mc = 0.0265, Table 3). This gene encodes a sodium/potassium/calcium exchanger (*NCKX1*), known primarily for its role in retinal photoreceptor function and calcium homeostasis. This gene has been reported as INF-stimulated in bats [51], highlighting its potential involvement in antiviral responses. While its specific function in immune cells remains to be fully elucidated, the induction of *SLC24A1* expression in response to interferon signaling indicates that it may participate in the modulation of immune responses, including those elicited by rubella vaccination. Perhaps its involvement in calcium homeostasis may influence B cell response, since calcium flux is crucial for B cell activation and differentiation. This highlights *SLC24A1* as a potentially significant biomarker of immune response, requiring further investigation into its role and effects in human immunity.

*TPT1P6* expression (Day 28–Day 0) change exhibited a negative association with the immune outcome (mc = −0.0435, Table 3). *TPT1P6* is a pseudogene (non-coding RNA) related to the translationally controlled tumor protein (*TPT1*, also known as *TCTP*). *TPT1*/*TCTP* has demonstrated cytokine-like functions in humans and enhancement of B cell proliferation in mouse models, suggesting it may influence B cell functions and response to stimuli [52,53]. Although pseudogenes like *TPT1P6* do not encode proteins, they may regulate gene expression post-transcriptionally, and *TPT1P6* could, therefore, potentially serve as a modulator of processes/pathways critical to B cell fate in the context of vaccination.

*RP11-51F16.9* expression (Day 28–Day 0) change exhibited a strong positive association with the immune outcome (mc = 0.1909, Table 3). It is a long non-coding RNA (lncRNA) that remains uncharacterized in the literature. Given the increasing recognition of lncRNAs in fine-tuning adaptive immune responses [54,55], the consistent association of *RP11-51F16.9* with rubella-specific memory B cell outcomes suggests it may be involved in transcriptional programs supporting the establishment of immunological memory.

Together, these genes play diverse roles, such as ionic regulation (*SLC24A1*), iron metabolism and viral entry (*TFR2*), and non-coding RNA-mediated gene expression control (*TPT1P6* and *RP11-51F16.9*), including potential modulation of immune responses (*TPT1P6*). This suggests that effective vaccine responses depend on a complex interplay of systems-level cellular regulation. The fact that these genes were significant at baseline and differentially expressed (for the Day 28–Day 0 gene expression modeling with memory B cell ELISpot response) following vaccination may indicate that pre-vaccination transcriptional profiles could serve as biomarkers for the subsequent RV-specific memory B cell response. Further investigation is required to elucidate the functional roles of these genes in B cell memory response, including whether their expression is directly or indirectly involved in B cell differentiation after antigen exposure.

### 3.4. Strengths and Limitations

The use of elastic-net linear regression enabled us to effectively handle such a high-dimensional dataset with multicollinearity-dependent data. The elastic-net penalty allowed for the identification of key predictor genes while avoiding overfitting by shrinking the coefficients, thus increasing the likelihood of identifying relevant genes when we have more predictors than samples.

While this study provides novel insights into transcriptional correlates of RV-specific humoral immunity following a third MMR dose, further functional characterization is needed. Future work should focus on validating the roles of candidate genes such as *TFR2*, *SLC24A1*, *TPT1P6*, and *RP11-51F16.9*, using in vitro models (i.e., siRNA knockdown or CRISPR-Cas9 approaches) and in vivo systems to confirm their contribution to B cell function and antibody responses.

A limitation of our study is the use of peripheral blood mononuclear cells (PBMCs), which represent a heterogeneous mixture of immune cell types, including B cells, T cells, monocytes, and natural killer cells. This cellular diversity may dilute transcriptomic signals and hinder the ability to attribute the identified gene expression signatures to specific cell populations. More specifically, PBMC heterogeneity may obscure cell-specific signals by averaging gene expression across multiple lineages, making it difficult to determine whether observed associations are primarily driven by B cells, T cells, or other subsets. Future studies employing single-cell transcriptomics or cell-type-specific gene expression, along with functional studies, could help identify the immune subsets responsible for the observed responses and refine the transcriptional factors and their associated specific cellular pathways critically modulating rubella vaccine-specific immunity.

## 4. Conclusions

This study provides new insights into transcriptional predictors of rubella-specific humoral immunity after a third MMR vaccine dose in women of childbearing age. We identified both baseline genes associated with key adaptive immune outcomes (IgG titer, neutralizing antibodies, and Day 28 memory B cell frequencies) and Day 28–Day 0 genes associated with Day 28 memory B cell frequencies. While the precise functional roles and mechanisms of action for each specific gene/factor remain to be elucidated, our findings suggest that transcriptional landscape at baseline (involved in cell adhesion, antigen processing and presentation, natural killer cell-mediated cytotoxicity, immunoglobulin production, and immune regulation) can shape immune responses to a third dose of MMR. Notably, transferrin receptor 2 (*TFR2*), a regulator of iron homeostasis and macrophage function, emerged as key determinant associated with several RV-specific immune outcomes, supporting its potential relevance in modulating rubella vaccine-induced immunity. Given its structural and functional similarity to other viral receptors, we speculate that *TFR2* may be involved in rubella virus binding and/or cell entry. Overall, this work underscores the potential for identifying transcriptional biomarkers and lays the groundwork for future investigations to inform personalized vaccination strategies, optimize booster schedules, and enhance vaccine efficiency in populations with variable immune responsiveness.

## Figures and Tables

**Table 1:** Elastic-net model results for the effect of baseline gene expression on rubella-specific immune outcomes after MMR vaccination. This table shows baseline genes associated with rubella virus-specific change (Day 28–Day 0) in (**A**) IgG antibody titer, (**B**) neutralizing antibody titer (reported as Karber NT), or (**C**) Day 28 RV-specific memory B cell frequencies (measured in memory B cell ELISpot) following a third dose of MMR vaccine that remained in the model.

	Gene Symbol	Gene Description	Coefficient ^1^
**A. Baseline genes associated with Day 28–Day 0 rubella IgG titer**	**SPSB1**	**splA/ryanodine receptor domain and SOCS box containing 1**	**0.1024**
	RP1-292B18.1	RP1-292B18.4	0.0800
	SLC25A1P5	solute carrier family 25 member 1 pseudogene 5	0.0686
	**SLC6A16**	**solute carrier family 6, member 16**	**0.0678**
	LRRC32	leucine rich repeat containing 32	0.0605
	HLA-DQB1	major histocompatibility complex, class II, DQ beta 1	0.0602
	DEGS1	delta 4-desaturase, sphingolipid 1	0.0519
	ZSCAN21	zinc finger and SCAN domain containing 21	0.0473
	**MTSS1**	**MTSS I-BAR domain containing 1**	**0.0383**
	CCNG2	cyclin G2	0.0303
	PPIAP29	peptidylprolyl isomerase A pseudogene 29	0.0293
	MEF2C	myocyte enhancer factor 2C	0.0290
	L3MBTL4	L3MBTL histone methyl-lysine binding protein 4	0.0187
	DUS2	dihydrouridine synthase 2	0.0178
	HILPDA	hypoxia inducible lipid droplet associated	0.0174
	CDKL1	cyclin-dependent kinase-like 1	0.0167
	VAV2	vav guanine nucleotide exchange factor 2	0.0158
	UBXN2A	UBX domain protein 2A	0.0144
	KIF20B	kinesin family member 20B	0.0126
	H2AFY2	H2A histone family, member Y2	0.0109
	POMZP3	POM121 and ZP3 fusion	0.0094
	**SDK2**	**sidekick cell adhesion molecule 2**	**0.0058**
	TIMM22	translocase of inner mitochondrial membrane 22	0.0031
	NUDT4	nudix hydrolase 4	0.0023
	**TPM3P6**	**tropomyosin 3 pseudogene 6**	**−0.0007**
	LDOC1	LDOC1 regulator of NFKB signaling	−0.0010
	HSPA6	heat shock protein family A (Hsp70) member 6	−0.0031
	AL390877.1	AL390877.1	−0.0048
	DHCR24	24-dehydrocholesterol reductase	−0.0049
	MSC	musculin	−0.0100
	GZMB	granzyme B	−0.0103
	RHOBTB3	Rho related BTB domain containing 3	−0.0133
	RNF5P1	ring finger protein 5 pseudogene 1	−0.0150
	CPA3	carboxypeptidase A3	−0.0221
	GBP5	guanylate binding protein 5	−0.0271
	**RAB38**	**RAB38, member RAS oncogene family**	**−0.0412**
	HLA-DQB2	major histocompatibility complex, class II, DQ beta 2	−0.0504
	ALOX5AP	arachidonate 5-lipoxygenase activating protein	−0.0641
	NOC4L	nucleolar complex associated 4 homolog	−0.0650
	RP11−209A2.1	RP11-209A2.1	−0.0754
	**TFR2**	**transferrin receptor 2**	**−0.0988**
	**SARS2**	**seryl-tRNA synthetase 2, mitochondrial**	**−0.0997**
	ZDHHC4	zinc finger DHHC-type palmitoyltransferase 4	−0.1141
	HLA-C	major histocompatibility complex, class I, C	−0.1158
	**PRR13P5**	**proline rich 13 pseudogene 5**	**−0.1370**
	RP11-216M21.1	RP11-216M21.1	−0.1529
**B. Baseline genes associated with Day 28–Day 0 rubella neut. Ab titer**	ZNF829	zinc finger protein 829	0.1104
	**MTSS1**	**MTSS I-BAR domain containing 1**	**0.0860**
	TBX19	T-box transcription factor 19	0.0855
	NUDT19	nudix hydrolase 19	0.0434
	C12orf5	TP53 induced glycolysis regulatory phosphatase	0.0297
	DUSP7	dual specificity phosphatase 7	0.0274
	GPRC5B	G protein-coupled receptor class C group 5 member B	0.0246
	**SPSB1**	**splA/ryanodine receptor domain and SOCS box containing 1**	**0.0211**
	**SLC6A16**	**solute carrier family 6 member 16**	**0.0203**
	SYCE1L	synaptonemal complex central element protein 1 like	0.0201
	BIRC2	baculoviral IAP repeat containing 2	0.0113
	QDPR	quinoid dihydropteridine reductase	0.0039
	GAN	gigaxonin	0.0018
	ARHGAP9	Rho GTPase activating protein 9	−0.0002
	ADCK4	coenzyme Q8B	−0.0012
	CBY1	chibby 1, beta catenin antagonist	−0.0029
	**RAB38**	**RAB38, member RAS oncogene family**	**−0.0052**
	DTNBP1	dystrobrevin binding protein 1	−0.0125
	KIR2DL4	killer cell immunoglobulin like receptor, two Ig domains and long cytoplasmic tail 4	−0.0143
	SERPINH1	serpin family H member 1	−0.0166
	CCNB1	cyclin B1	−0.0222
	TMC4	transmembrane channel like 4	−0.0235
	DNASE1L3	deoxyribonuclease 1L3	−0.0242
	RUVBL2	RuvB like AAA ATPase 2	−0.0253
	TPRA1	transmembrane protein adipocyte associated 1	−0.0312
	**TPM3P6**	**tropomyosin 3 pseudogene 6**	**−0.0344**
	LGALS9B	galectin 9B	−0.0503
	RP11-360D2.2	RP11-360D2.2	−0.0509
	CLSTN3	calsyntenin 3	−0.0644
	**TFR2**	**transferrin receptor 2**	**−0.0890**
	PRUNE2	prune homolog 2 with BCH domain	−0.1028
	**SARS2**	**seryl-tRNA synthetase 2, mitochondrial**	**−0.1043**
	CHCHD2	coiled-coil-helix-coiled-coil-helix domain containing 2	−0.1125
	ABCG1	ATP binding cassette subfamily G member 1	−0.1149
	**PRR13P5**	**proline rich 13 pseudogene 5**	**−0.1629**
**C. Baseline genes associated with Day 28 RV-specific MBC frequencies**	C17orf89	NADH: ubiquinone oxidoreductase complex assembly factor 8	0.1308
	TPT1P6	TPT1 pseudogene 6	0.0968
	MTPAP	mitochondrial poly(A) polymerase	0.0594
	RP11-100N21.1	RP11-100N21.1	0.0388
	SLC35E2	solute carrier family 35 member E2A (pseudogene)	0.0256
	EGLN3	egl-9 family hypoxia inducible factor 3	0.0074
	IMMT	inner membrane mitochondrial protein	−0.0004
	ARHGEF19	Rho guanine nucleotide exchange factor 19	−0.0021
	MTHFSD	Methenyltetrahydrofolate synthetase domain containing	−0.0063
	FUZ	fuzzy planar cell polarity protein	−0.0152
	C16orf93	cilia and flagella associated protein 119	−0.0177
	FAM47E-STBD1	FAM47E-STBD1 readthrough	−0.0212
	IGFLR1	IGF like family receptor 1	−0.0279
	SLC24A1	solute carrier family 24 member 1	−0.0317
	**SDK2**	**sidekick cell adhesion molecule 2**	**−0.0534**
	**TFR2**	**transferrin receptor 2**	**−0.0773**
	ENOX2	ecto-NOX disulfide-thiol exchanger 2	−0.0922
	SLC25A20	solute carrier family 25 member 20	−0.0948
	RP11-51F16.9	RP11-51F16.9	−0.1371

^1^ Positive coefficients indicate that higher gene expression is associated with an increase in the immune outcome, and likewise, lower levels of gene expression are associated with a decrease in the immune outcome. Bold indicates that the gene has appeared in modeling for two or more different immune outcomes.

**Table 2:** Genes predictive of two or more outcomes.

Gene	Coefficient ^1^	Prediction
MTSS1	0.038	Baseline gene predictive of change (Day 28–Day 0) in rubella IgG titer
	0.086	Baseline gene predictive of change (Day 28–Day 0) in neutralizing antibodies
PRR13P5	−0.137	Baseline gene predictive of change (Day 28–Day 0) in rubella IgG titer
	−0.163	Baseline gene predictive of change (Day 28–Day 0) in neutralizing antibodies
RAB38	−0.041	Baseline gene predictive of change (Day 28–Day 0) in rubella IgG titer
	−0.005	Baseline gene predictive of change (Day 28–Day 0) in neutralizing antibodies
SARS2	−0.100	Baseline gene predictive of change (Day 28–Day 0) in rubella IgG titer
	−0.104	Baseline gene predictive of change (Day 28–Day 0) in neutralizing antibodies
SDK2	0.006	Baseline gene predictive of change (Day 28–Day 0) in rubella IgG titer
	−0.053	Baseline gene predictive of Day 28 memory B cell frequency
SPSB1	0.102	Baseline gene predictive of change (Day 28–Day 0) in rubella IgG titer
	0.021	Baseline gene predictive of change (Day 28–Day 0) in neutralizing antibodies
TPM3P6	−0.001	Baseline gene predictive of change (Day 28–Day 0) in rubella IgG titer
	−0.034	Baseline gene predictive of change (Day 28–Day 0) in neutralizing antibodies
SLC6A16	0.068	Baseline gene predictive of change (Day 28–Day 0) in rubella IgG titer
	0.020	Baseline gene predictive of change (Day 28–Day 0) in neutralizing antibodies
	−0.001	(Day 28–Day 0) gene expression associated with Day 28 memory B cell frequency
TFR2	−0.099	Baseline gene predictive of change (Day 28–Day 0) in rubella IgG titer
	−0.089	Baseline gene predictive of change (Day 28–Day 0) in neutralizing antibodies
	−0.077	Baseline gene predictive of Day 28 memory B cell frequency
	0.003	(Day 28–Day 0) gene expression associated with Day 28 memory B cell frequency

^1^ Positive coefficients indicate that higher gene expression is associated with an increase in the immune outcome, and likewise lower levels of gene expression are associated with a decrease in the immune outcome.

**Table 3:** Elastic-net model results for the effect of (Day 28–Day 0) gene expression associated with Day 28 rubella virus-specific memory B cell frequencies. This table shows (Day 28–Day 0) genes associated with Day 28 rubella virus-specific memory B cell frequencies (measured in memory B cell ELISpot) following a third dose of MMR vaccine.

Gene Symbol	Gene Description	Coefficient ^1^
RP11-51F16.9	RP11-51F16.9	0.1909
KATNB1	katanin regulatory subunit B1	0.0375
TIGIT	T cell immunoreceptor with Ig and ITIM domains	0.0271
SLC24A1	solute carrier family 24 member 1	0.0265
FBXO4	F-box protein 4	0.0177
ALG8	ALG8 alpha-1,3-glucosyltransferase	0.0176
RP11-886H22.1	RP11-886H22.1	0.0099
SERBP1P5	SERPINE1 mRNA binding protein 1 pseudogene 5	0.0074
**TFR2**	**transferrin receptor 2**	**0.0031**
**SLC6A16**	**solute carrier family 6 member 16**	**−0.0013**
PEX11G	peroxisomal biogenesis factor 11 gamma	−0.0031
PPAP2A	phospholipid phosphatase 1	−0.0108
CSAD	cysteine sulfinic acid decarboxylase	−0.0237
IRF1	interferon regulatory factor 1	−0.0282
FBRS	fibrosin	−0.0430
TPT1P6	TPT1 pseudogene 6	−0.0435
RECQL4	RecQ like helicase 4	−0.0671
EFCAB10	EF-hand calcium binding domain 10	−0.0734

^1^ Positive coefficients indicate that higher gene expression is associated with an increase in the immune outcome, and likewise, lower levels of gene expression are associated with a decrease in the immune outcome. Bold indicates that the gene has appeared in modeling for two or more different immune outcomes.

## Data Availability

The datasets presented in this article are not readily available because the data are part of an ongoing study. Requests to access the datasets should be directed to Richard B. Kennedy (kennedy.rick@mayo.edu).

## Abbreviations

The following abbreviations are used in this manuscript:

CRS	Congenital rubella syndrome
ELISA	Enzyme-linked immunosorbent assay
ICC	Intra-class correlation coefficient
IgG	Immunoglobulin G
lncRNA	Long non-coding RNA
mc	Model coefficient
mRNA	Messenger ribonucleic acid
NGS	Next-generation sequencing
PBMCs	Peripheral blood mononuclear cells
Q1	First quartile (25th percentile)
Q3	Third quartile (75th percentile)
RNA	Ribonucleic acid
RV	Rubella virus
SFUs	Spot forming units
TLR	Toll-like receptor

## Appendix A

### Demographics and Immune Response Characterization of the Study Subjects

**Table A1:** Demographic and screening of the study subjects.

	Low (n = 53)	High (n = 45)	Total (n = 98)
**Sex**			
Female	53 (100.0%)	45 (100.0%)	98 (100.0%)
**Race**			
Asian	0 (0.0%)	1 (2.2%)	1 (1.0%)
White	53 (100.0%)	44 (97.8%)	97 (99.0%)
**Ethnicity**			
Non-Hispanic nor Latino	51 (96.2%)	44 (97.8%)	95 (96.9%)
Hispanic or Latino	2 (3.8%)	1 (2.2%)	3 (3.1%)
**Age at enrollment/3rd MMR vaccine dose (years)**			
Median	35.7	33.9	35.2
Q1, Q3	31.4, 40.3	30.4, 40.9	30.7, 40.4
Range	21.5–44.9	22.6–45.1	21.5–45.1
**Age at 1st rubella vaccination (months)**			
Median	15.8	15.4	15.7
Q1, Q3	15.1, 18.0	14.9, 16.0	15.0, 17.0
Range	11.9–352.9	4.6–312.3	4.6–352.9
**Age at 2nd rubella vaccination (years)**			
Median	12.2	12.4	12.2
Q1, Q3	9.8, 16.6	11.0, 17.2	10.1, 17.0
Range	4.3–35.1	1.5–28.3	1.5–35.1
**Time from 2nd rubella vaccination to enrollment/3rd MMR vaccine dose (years)**			
Median	23.2	22.7	23.2
Q1, Q3	19.9, 25.1	18.0, 26.3	18.7, 25.5
Range	1.9–39.0	3.9–40.6	1.9–40.6
**Prior rubella ELISA Ab titers ^1^**			
Median	0.2	1.3	0.3
Q1, Q3	0.1, 0.3	1.1, 1.6	0.2, 1.2
Range	0.1–0.3	0.8–3.4	0.1–3.4

Bold text indicates the heading for each demographic or clinical variable. ^1^ Prior rubella antibody titers were measured by ELISA and are expressed as optical density units.

**Table A2:** Immune response characterization of the study subjects.

	Low (n = 53)	High (n = 45)	Total (n = 98)
**Day 28–Day 0 Karber NT_50_ ^1^**			
N-Miss	1	3	4
Median	153.9	122.8	136.8
Q1, Q3	75.1, 269.3	68.9, 216.5	70.7, 246.1
**Day 28–Day 0 rubella IgG titer (IU/mL)**			
N-Miss	1	3	4
Median	95.1	49.3	66.0
Q1, Q3	46.8, 176.7	24.7, 84.2	36.3, 123.9
**Day 28-Day 0 avidity index (%)**			
N-Miss	4	3	7
Median	11.2	5.6	7.9
Q1, Q3	6.2, 16.4	2.6, 8.1	4.2, 13.6
**Day 28 memory B cell ELISpot (SFUs/200,000 cells/PBMCs) ^2^**			
N-Miss	10	12	22
Median	27.5	40.0	29.8
Q1, Q3	13.0, 43.8	18.5, 66.5	16.0, 50.6

Bold text indicates the heading for each demographic or clinical variable. ^1^ The neutralization titer represented the highest dilution at which the input virus signal was reduced by at least 50% within the dilution series (NT_50_). ^2^ ELISpot results are expressed in spot forming units (SFUs) per 200,000 peripheral blood mononuclear cells (PBMCs).
